# Effects of Acetabular Screws on the Initial Stability of Porous Coated Acetabular Implants in Revision Total Hip Arthroplasty

**DOI:** 10.7759/cureus.38257

**Published:** 2023-04-28

**Authors:** Nathan B Kaplan, Matthew F Barra, Ayodeji Jubril, Emma Knapp, Hani Awad, John Ginnetti

**Affiliations:** 1 Department of Orthopaedics and Rehabilitation, University of Rochester Medical Center, Rochester, USA; 2 Department of Orthopaedics and Rehabilitation/Center for Musculoskeletal Research, University of Rochester Medical Center, Rochester, USA; 3 Department of Orthopaedics and Rehabilitation/Center for Musculoskeletal Research/Department of Biomedical Engineering, University of Rochester Medical Center, Rochester, USA

**Keywords:** paprosky iib, revision total hip arthroplasty, initial stability, screw fixation, acetabular bone loss

## Abstract

Introduction: Revision total hip arthroplasty in the setting of acetabular bone loss remains a challenging clinical entity. Deficiencies of the acetabular rim, walls, and/or columns may limit the bony surface area and initial acetabular construct stability necessary for osseointegration of cementless components. Press-fit acetabular components with supplemental acetabular screw fixation represent a common technique aimed to minimize implant micromotion and allow for definitive osseointegration. Although acetabular screw fixation is commonly practiced in revision hip arthroplasty, few studies to date have examined the screw properties associated with maximal acetabular construct stability. The purpose of the present report is to examine acetabular screw fixation in a pelvis model mimicking Paprosky IIB acetabular bone loss.

Methods: Measuring bone-implant interface micromotion as a surrogate for initial implant stability, experimental models assessed the effect of screw number, screw length, and screw position on construct stability subject to a cyclic loading protocol designed to replicate joint reaction forces of two common daily activities.

Results: Trends towards increasing stability were demonstrated with increasing screw number, increasing screw length, and concentrating screws in the supra-acetabular dome. All experimental constructs yielded micromotion levels sufficient for bone ingrowth, except when screws in the dome were moved to the pubis and ischium.

Conclusions: When using a porous coated revision acetabular implant to treat Paprosky IIB defects, screws should be used, and furthermore, increasing number, length, and position within the acetabular dome may help further stabilize the construct.

## Introduction

Porous-coated total hip acetabular components offer the advantage of stable, long-term fixation in primary and revision total hip arthroplasty. Osseointegration, the process of host subchondral bone incorporating into a porous metal surface, is required to establish enduring fixation of cementless acetabular implants. Successful osseointegration demands both intimate contact of the porous acetabular implant surface with host bone of sufficient quality and quantity as well as initial acetabular implant stability historically defined as micromotion of less than 150 µm [1-3]. Acetabular bone loss challenges the biologic as well as the biomechanical prerequisites of osseointegration. Deficiencies of acetabular bone may not only limit the bony surface area available to the well-positioned acetabular implant but also generate increased strain across the bone-implant interface, thus inhibiting biologic bone ingrowth. Implant design modifications including construct shape, surface topography, and porous coating features have sought to stabilize the bone-acetabular implant articulation and promote osseointegration in the absence of supplemental fixation. However, the press-fit impaction of a porous coated acetabular implant with augmented acetabular screw fixation is generally regarded as the most rigid construct [1,4].

Unfortunately, the use of supplemental acetabular screw fixation in the setting of acetabular bone loss does not eliminate aseptic cup loosening from the aftermath of surgery. This persistent threat of mechanical acetabular failure motivates orthopaedic surgeons to think critically about screw placement during the time of revision arthroplasty in order to optimize initial implant stability. The Paprosky type IIB acetabular defect, characterized by substantial loss of the superolateral acetabular rim with intact and supportive acetabular columns, is considered the most common yet severe bone defect in which an uncemented revision acetabular component can be utilized with strictly acetabular screw supplemental fixation [5]. In an effort to objectively define optimal acetabular screw characteristics, we sought to quantify the differential stability of acetabular constructs in a Sawbones model simulating the Paprosky Type IIB acetabular defect, while systematically varying the number, length and location of screw placement. The goal of this study was to evaluate the effect of altering three different screw variables (length, number, and position) on micromotion at the bone-implant interface, in the simulated setting of Paprosky type IIB acetabular bone loss. To our knowledge, no biomechanical study has addressed this question in a revision model with acetabular bone loss.

## Materials and methods

**Figure 1:**
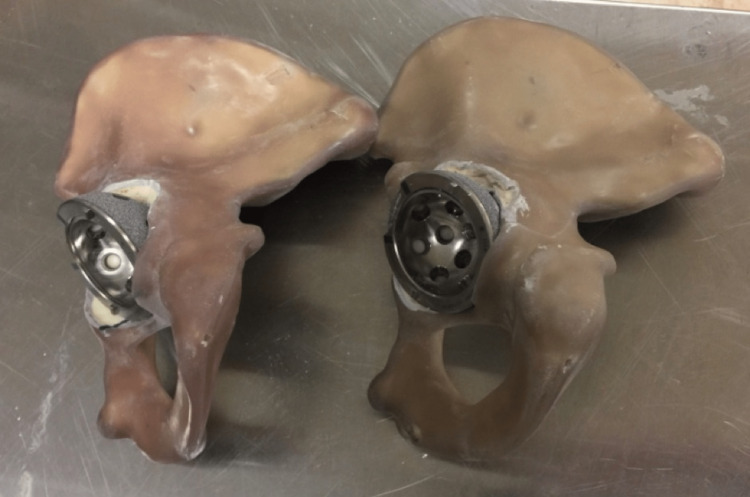
Sawbones composite bone hemi-pelvis models Sawbones composite bone hemi-pelvis models with simulated Paprosky type IIB acetabular defects and press-fit highly porous multi-hole revision acetabular components.

In this study, we used Sawbones fourth Generation Composite hemi-pelvis models (Sawbones, model #3405; Pacific Research Laboratories, Vashon, WA) that were fashioned to simulate acetabular bone loss consistent with Paprosky type IIB acetabular defects. Models were created by sequentially over reaming the acetabulum, and removing the posterior-superior aspect of the acetabular rim with a sagittal saw (Figure 1). A revision acetabular component (Zimmer-Biomet Trabecular Metal revision acetabular shell; Zimmer-Biomet, Warsaw, IN) was then implanted with using anatomic landmarks to position the component within the Lewinnick safe zone of abduction and anteversion. Impacted acetabular shells achieved partial intrinsic stability.

**Figure 2:**
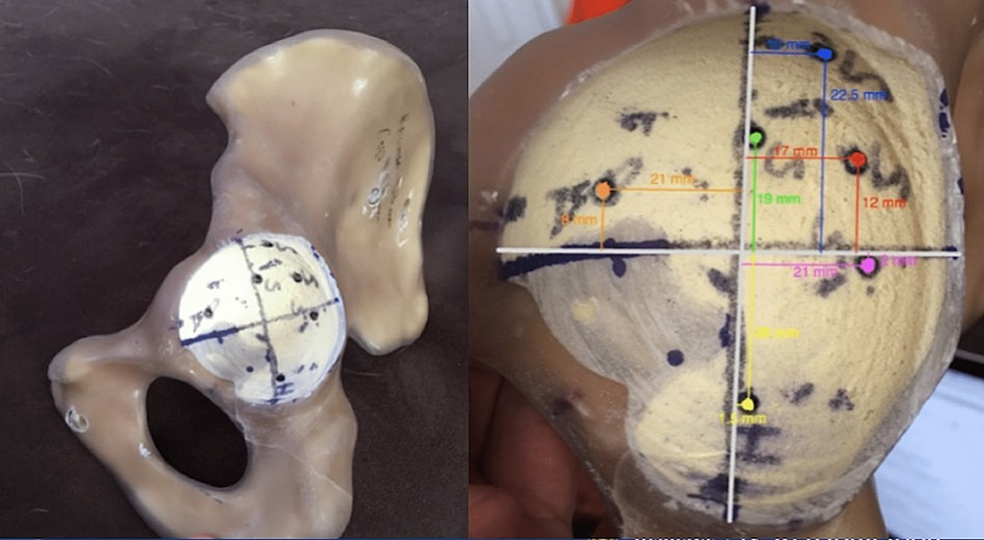
Standardized screw positions established using anatomic screw safe-zones.

A two-by-two grid was drawn in the acetabular cavity using anatomic landmarks to map screw position safe zones (Figure 2). Screw positions were standardized between models using this grid, with four supra-acetabular screw positions inside or just outside of the posterior-superior target zone quadrant. Two supplemental screw positions were mapped: one in the pubic eminence (anterior-inferior quadrant), and one in the ischium (posterior-inferior quadrant). Next, 6.5 mm self-tapping titanium bone screws (Zimmer-Biomet, Warsaw, IN) compatible with the acetabular component were used as outlined in the experimental groupings.

**Figure 3:**
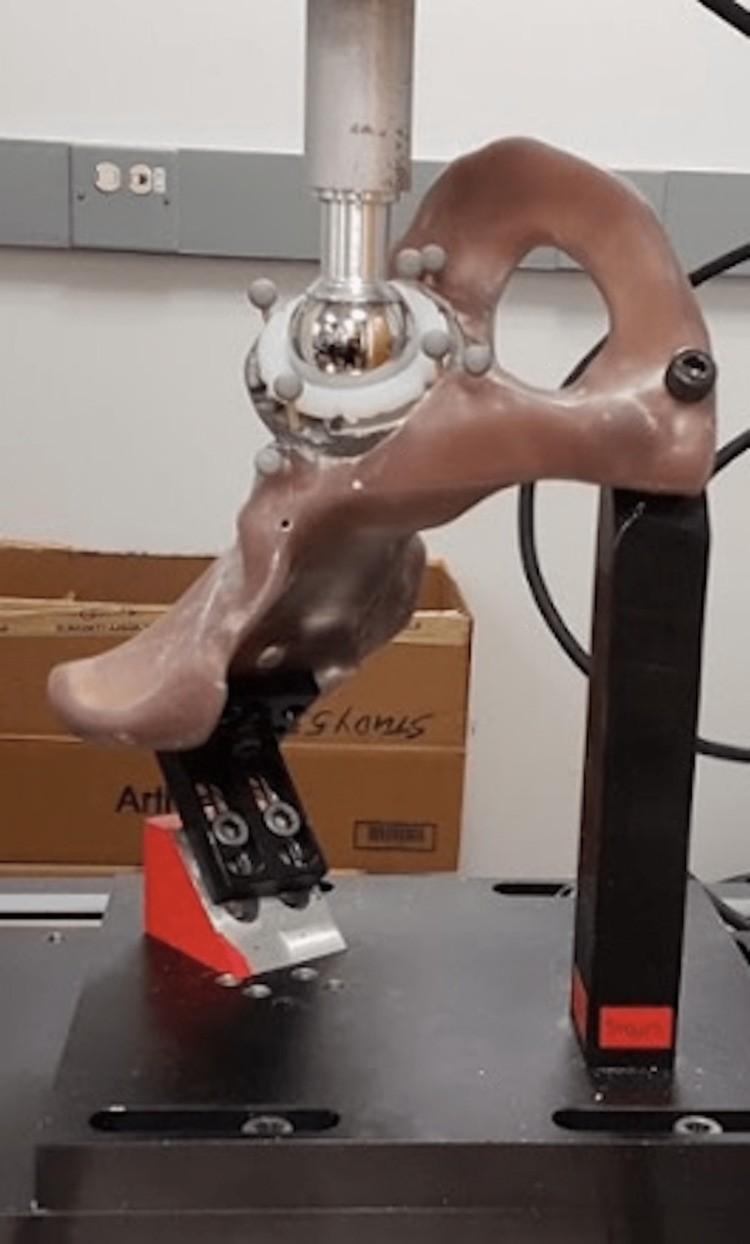
A cyclic loading apparatus using two different custom machined mounts Cyclic loading apparatus using two different custom machined mounts designed to replicate joint reactive force vectors consistent with vectors described by Bergmann et al. [6] for walking on flat ground and descending stairs. Reference markers for the OptiTrack motion capture system (OptiTrack Prime13, OptiTrack Motive software, NaturalPoint Inc., Corvallis, OR) were positioned on the component and surrounding acetabular bone.

Hemi-pelvis specimens were then mounted into a linear load cell (ElectroPuls E10000, Instron, Norwood, MA) using two custom machined mounts that were each fabricated to simulate an applied force vector consistent with previously defined joint reaction forces for two common daily activities: walking on flat ground, and descending stairs (Figure 3). These force vectors were previously defined by Bergmann et al. in their study of hip joint reaction forces from routine activities [6]. These two activities were chosen to represent the most common (walking) and the highest magnitude (descending stairs) forces encountered by hip prostheses in daily activities as identified by Bergmann et al. Each specimen underwent a cyclic loading protocol of 175 cycles under both activity vectors in randomized order, as used in a previous study evaluating implant stability in a pelvic discontinuity model by Gilliland et al. [7]. The loading protocol involved sinusoidal loading to a peak of 1900 N at a rate of 0.5 Hz. The first 25 cycles of each protocol were discarded to allow for preconditioning of the specimen and settling of the implant.

The primary endpoint, micromotion at the bone-implant interface, was measured using a motion capture system (OptiTrack Prime13, OptiTrack Motive software, NaturalPoint Inc., Corvallis, OR) with a manufacturer’s reported precision of 5-10 mm. Motion capture reference markers were positioned on the rim of the component and on the acetabular bone to determine motion of the component in three planes: posterolateral rotation (XY) into the rim defect, anteromedial rotation (YZ), and horizontal rotation (XZ) around the rim equator of the component. Displacement over time was recorded in these three planes of motion using a custom MatLab equation, and force-displacement curves were created. After determining the displacement per load cycle in each of the three defined planes of motion, the total motion of a point on the spherical surface of the implant was estimated using a calculation for motion of a point on a sphere.

Experimental specimens were divided into groups based on three screw variables: number, length, and position. Additionally, a control group in which no screws were implanted was created to assess motion without screw supplementation. Screw number was varied between two, three, and four 25 mm length screws in the supra-acetabular position. Screw length was varied utilizing two screws of 25- and 50-mm lengths used in the supra-acetabular position. Screw position was varied between three screws used in supra-acetabular, anterior (pubic), and posterior (ischial) positions. Three specimens were tested in each control and experimental group. Micromotion per load cycle was recorded in each plane of motion, and from these measurements, the total motion was calculated for each specimen.

ANOVA comparative statistics were then run between each control and experimental group to establish statistical significance between differences in displacement, with a p<0.05. As previously defined and referenced in several studies, a threshold of >150 µm of motion was established as the point over which, fibrous ingrowth would be expected. A priori power analysis was not performed as no study has been published using this method of measurement of micromotion on the surface of an acetabular component, and therefore benchmark effect sizes are not available.

## Results

With three specimens in each control and experimental group, a total of 36 models were tested. Table 1 demonstrates the mean micromotion (mm) in each group. In the majority of models, the largest magnitude of displacement occurred in the posterolateral plane (XY). Therefore, in observing the trends in micromotion in each group, we elected to focus on this plane of motion and the total motion when analysing the results.

**Table 1:** Mean implant micromotion in µm (SD) SA: Superoanterior, Ant: Anterior, Post: Posterior.

	XY plane	YZ plane	XZ plane	Total
Screw Number				
No Screws	258.67 (63.56)	120.83 (13.25)	69.17 (7.52)	294.42 (60.72)
Screws x2	59.50 (31.85)	77.33 (15.82)	40.83 (25.91)	107.08 (38.96)
Screws x3	66.00 (32.11)	67.67 (3.33)	89.67 (22.26)	133.84 (11.32)
Screws x4	61.83 (25.08)	67.33 (13.58)	65.33 (19.75)	115.00 (17.47)
Screw Position				
SA x3	66.00 (32.11)	67.67 (3.33)	89.67 (22.26)	133.84 (11.32)
SA x2, Ant	115.83 (11.73)	52.17 (13.55)	29.67 (4.19)	130.95 (12.11)
SA x2, Post	121.50 (19.37)	72.17 (41.33)	35.00 (10.44)	150.45 (5.77)
SA, Ant, Post	141.33 (60.66)	85.00 (21.93)	34.00 (13.11)	173.93 (38.62)
Screw Length				
25 SA x2	59.50 (31.85)	77.33 (15.82)	40.83 (25.91)	107.08 (38.96)
25 Ant, 50 Post	89.17 (15.25)	78.00 (21.66)	51.33 (15.50)	131.45 (5.30)
50 Ant, 25 Post	76.17 (14.00)	131.50 (27.83)	48.00 (5.07)	159.97 (26.57)
50 SA x2	52.67 (25.47)	105.83 (13.61)	47.17 (21.83)	130.59 (5.25)

**Figure 4:**
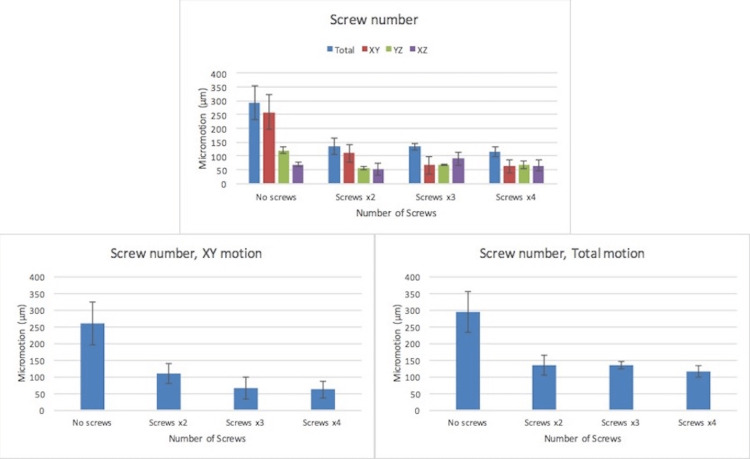
Implant micromotion (µm) as a function of screw number in all three planes of recorded motion and calculated total motion

There was a trend towards decreased cup motion as screw number increased. This trend was observed in both the XY plane of motion and when total motion was calculated (Figure 4); however, it was not observed in all motion planes. The trend was statistically insignificant. The addition of two or more screws reduced overall motion below the threshold of 150 µm.

**Figure 5:**
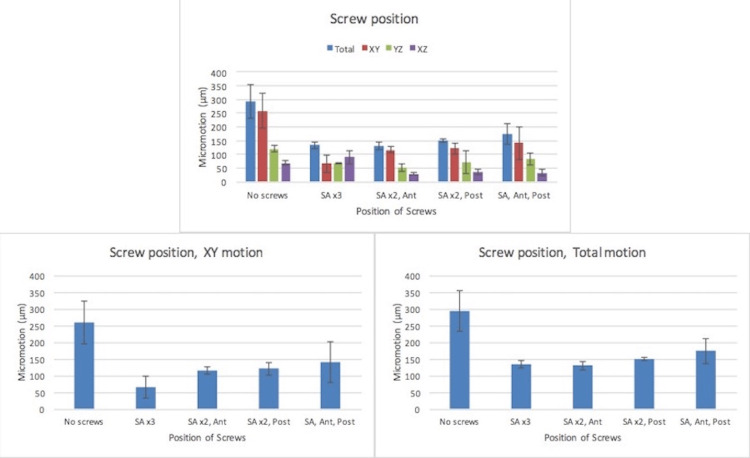
Implant micromotion (µm) as a function of screw position in all three planes of recorded motion and calculated total motion SA: Superoanterior, Ant: Anterior, Post: Posterior.

There was a trend towards increased cup motion when screws were moved from the supra-acetabular position to either an anterior or posterior position. Again, this trend was observed in both the XY plane of motion and when total motion was calculated (Figure 5). This trend, was also statistically insignificant between groups. When total motion is considered, moving two screws from the supra-acetabular position into anterior and posterior positions, the overall motion is above the threshold for ingrowth. Motion was below threshold for all other groups when assessing screw position.

**Figure 6:**
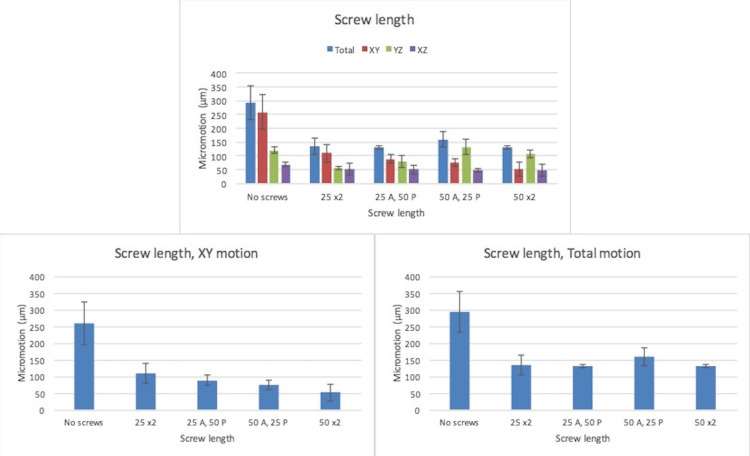
Implant micromotion (µm) as a function of screw length in all three planes of recorded motion and calculated total motion A: Anterior, P: Posterior.

There was a trend towards decreased cup motion as screw length was increased. In general, this trend was observed in both the XY plane and total motion; however, there was an unusually high average motion in the group with a 50 mm screw designated A and 25 mm screw designated P in the YZ plane, and this led to a higher total calculated motion in this group (Figure 6). Once again, this trend was not statistically significant between experimental groups. The addition of screws in all experimental groups dropped cup motion below the threshold for ingrowth.

While the trends observed in the experimental groups were not statistically significant between the experimental groups, there was a statistically significant decrease in micromotion from the control group with no screws to each of the experimental screw groups.

## Discussion

Stable arthroplasty component fixation is paramount for the long-term survivorship of cementless implants in primary and revision hip arthroplasty. During hip arthroplasty, the absence of supportive acetabular bone secondary to osteolysis, overaggressive acetabular reaming, dysplastic acetabular morphology, previous acetabular fracture, and/or advanced avascular necrosis may jeopardize the surgeon’s ability to achieve stable non-augmented acetabular fixation. When full intrinsic stability of the acetabular shell cannot be obtained, options for supplemental fixation include acetabular screws, porous metal augments, allograft/autograft bone, cement, reconstruction cages, and custom triflange devices. Utilization of increasingly sophisticated reconstruction devices is not only costly but frequently requires larger surgical exposures and increased operative times, two variables that are undoubtedly associated with an increased risk of periprosthetic joint infection. Thus, multi-hole acetabular shells in combination with acetabular screws remain an attractive technique within the surgeon armamentarium. A paucity of objective evidence exists to guide surgeon placement of acetabular screws. Several previous studies have demonstrated the long-term reliability of porous coated implants using a press-fit technique and supplemental screw fixation in the revision setting as an alternative to implants with pegs, spikes, and dual geometries [4,8-11]. In the classic report, Wasielewski et al. has provided anatomical parameters for acetabular screw placement based on the location of adjacent neurovascular structures [12]. Still, important biomechanical questions regarding optimal screw length, number, location, and configuration have remained largely unanswered.

In this study, we were able to identify trends in decreasing micromotion, and therefore, increased initial stability of a cementless component with increasing screw length, screw number, and concentrating screws in a superior location within the acetabulum. Changing screw position (and thus, increasing divergence), led to a decrease in stability, despite prior evidence to suggest that screw divergence can increase construct stability [13]. Given our experimental design, this contradictory finding may be explained by a tethering effect of screws against the largest displacement vector imparted on our component constructs. In the Paprosky IIB defect bone loss pattern characterized by substantial loss of the superolateral acetabular rim, the greatest displacement vector was rotation into the superior and lateral acetabular defect. Interestingly, in specimens with anterior and posterior screw placement, the anterior and posterior screws were found to have loosened significantly after loading. Therefore, a plausible alternative explanation is superior screws resist the largest displacement vector during early loading, and following superior screw removal the constructs were rendered vulnerable to subsequent cyclical loading. Further testing is required to assess the benefit of pubic and/or ischial screws in this model.

The threshold of micromotion, which allows for ossoeintegration of the porous coated implant, varies by published historical report. In one of the earliest studies, Pilliar et al., evaluated the effect of micromotion on ingrowth of porous metal in a linear displacement model. This nascent report demonstrated that excess movement (>150 µm) results in fibrous ingrowth rather than bone ingrowth [3]. Consequently, 150 µm is a frequently cited threshold in subsequent biomechanical studies evaluating the stability of acetabular components [14]. Surprisingly, few reports have measured micromotion at the implant-bone interface in a non-linear model. To date, there is no standard for reporting motion in a revision hip model in which cup displacement is both rotational and linear [7,14]. In the present report, with exception of the “divergent” specimen (specimen with three total screws, one screw superior, one screw anterior, one screw posterior), addition of at least two screws to each specimen led to a statistically significant decrease in micromotion below the 150 µm threshold in all planes of motion, including our calculated total motion.

Several weaknesses of our report are acknowledged. First, we elected to use sawbones models, as opposed to cadaveric specimens, in an effort to maximize the number of specimens and standardize experimental groups. While the biomechanical composite Sawbones models may provide a basis for better understanding bone implant interactions, sawbones models fail to replicate the pathologic state of acetabular bone frequently encountered during revision arthroplasty in the setting of bone loss and therefore these results may differ if studied in vivo. Additionally, although we focused on one acetabular bone loss pattern, understanding that in vivo bone loss patterns are neither predictable nor identical. Further study of additional bone loss patterns may further elucidate screw locations, which more effectively counter motion vectors corresponding to specific regions of acetabular bone deficiency. Second, our applied loading protocol established by Bergmann et al., relies upon simplified linear vectors, which may not accurately represent the physiologic joint reactive forces transmitted through the acetabular component-bone interface [6,15]. Third, our experimental methodology intentionally limited experimental test conditions (i.e., 25 mm screws in all groups, longest screw possible anterior screw) and selection of single size elliptical Zimmer Biomet TM implant to minimize the variability between groups. Therefore, vast opportunity exists to expand upon the current study design. Fourth, despite the observed trends, the limited effect size noted may not be clinically relevant. Fifth, the screw trajectories examined in this study represent distinct anatomical planes and this must be recognized if applying these principles in patients with altered pelvic parameters (i.e., scoliosis patients with altered pelvic tilt or obliquity). Sixth, despite using a porous coated acetabular cup which is designed to optimize biologic fixation of the cup (in-growth and on-growth), this study only considers the mechanical factors related to cup stability. Despite this, the mechanical factors related to cup stability are vital determinants in overall integration to prevent excessive micromotion during the period prior to full osseointegration of the cup. However, it is likely that the type of cup used and its associated coating will have effects on long term clinical outcomes. Last, and perhaps most importantly, the trends observed in our experimental groups did not demonstrate statistical significance. We were unable to perform a priori power analysis, and it is possible that increasing the specimens in each group may allow these differences to reach statistical significance as the overall number of models tested resulted in a relatively small data set.

## Conclusions

The data presented in this study supports the widely held belief that porous coated acetabular implants inserted with the press-fit technique in combination with supplemental screw fixation generate the initial stability necessary for long-term stability via osseointegration in Paprosky IIB acetabular defects. Furthermore, increasing the screw number, length, and position within the acetabular dome may help further stabilize the construct. Together, these findings warrant surgeon consideration during real-time placement of acetabular screws in Paprosky IIB defects.
